# Molecular epidemiology and seroprevalence in asymptomatic *Plasmodium falciparum* infections of Malagasy pregnant women in the highlands

**DOI:** 10.1186/s12936-015-0704-5

**Published:** 2015-05-03

**Authors:** Oumou Maïga-Ascofaré, Raphael Rakotozandrindrainy, Mirko Girmann, Andreas Hahn, Njary Randriamampionona, Sven Poppert, Jürgen May, Norbert G Schwarz

**Affiliations:** Bernhard-Nocht Institute for Tropical Medicine, Hamburg, Germany; University of Antananarivo, Antananarivo, Madagascar; Justus-Liebig-University, Giessen, Germany

**Keywords:** Madagascar, Pregnancy, *Plasmodium falciparum*, Seroprevalence, Drug resistance, Multiplicity of infection

## Abstract

**Background:**

Malaria epidemiology in Madagascar is classified into four different areas, ranging from unstable seasonal transmission in the highlands to hyperendemic perennial transmission areas in the costal level. Most malaria studies in Madagascar are focused on symptomatic children. However, because of the low transmission in some areas with correspondingly low level of semi-immunity, adults are also at risk, in particular pregnant women. The objective of this study was to gain information on the genetic epidemiology of malarial infections in pregnant women in order to provide information for malaria control and elimination programmes in Madagascar.

**Methods:**

Between May and August 2010, we carried out cross-sectional surveys targeting healthy pregnant women in six locations, three in the coastal area and three in the highlands at 850–1300 m. 1244 blood samples were screened for anti-*Plasmodium falciparum* antibodies by immunofluorescence test and for malarial infection by realtime-PCR. The prevalence of chloroquine and sulphadoxine-pyrimethamine resistance markers was also determined in all *Plasmodium falciparum* samples by PCR-RFLP as well as the multiplicity of infection through genotyping six neutral microsatellites.

**Results:**

In the highlands, 67.4% of the women presented antibodies against *Plasmodium falciparum* and 9.2% were carrying parasites, at the coast 95.6% and 14.8%, respectively. In the mean, 1.2 clones were detected in infected pregnant woman in the highlands and 1.5 at the coast. A higher level of monoclonal infections was found in the highlands (85.4%) compared to the coast (61.8%). Resistance markers for sulphadoxine-pyrimethamine were present only in two sites.

**Conclusion:**

Immunity is triggered in Malagasy highland populations when they are infected with malaria parasites, but these populations could also serve as a reservoir for epidemics.

**Electronic supplementary material:**

The online version of this article (doi:10.1186/s12936-015-0704-5) contains supplementary material, which is available to authorized users.

## Background

Asymptomatic carriage of malaria parasites is common in adults in endemic regions and provides a reservoir for gametocytes, the forms of the parasite infectious to mosquitoes and thus responsible for cross-species transmission [1]. These asymptomatic carriers may be of particular importance in the highland regions of Madagascar, where malaria can be absent over years, but may reappear epidemically, like between 1986 and 1988 where malaria outbreaks had devastating effects on a virtually non-immune population [2,3]. Malaria parasites found on the island of Madagascar are *Plasmodium falciparum*, *Plasmodium vivax* and to a minor degree *Plasmodium malariae* and *Plasmodium ovale* [4]. The main malaria vector in the Madagascan highlands is *Anopheles funestus* that once had disappeared after extensive insecticide spraying in the 1950s, but reappeared in the 1980s [5] leading to epidemics that caused several ten thousand of deaths in the 1980s and 1990s [6].

The country has been classified in four distinct malaria epidemiologic zones based on the geography and the length and intensity of malaria transmission. Malaria transmission is stable and perennial at the East Coast, stable and long seasonal at the West Coast and unstable and seasonal in the Central Highlands and the Semi-desert zones of the South [4]. On the East and West Coast, exposure and thus immunity among adults is reported to be high and most morbidity and mortality is among children under five and pregnant women. In the upper Central Highlands and the South immunity is limited making the population vulnerable to epidemics especially during the rainy season (from late October/early November until May).

Since 2008, The National Malaria Control Programme (NMCP) of Madagascar received international support through funds from the Global Funds, the World Health Organization and the US President’s Malaria Initiative as help in their fight to eradicate malaria from the island [7]. Nevertheless, only little is known on the recent epidemiology of *P. falciparum* infections in the country [8,9]. Most studies were performed in symptomatic children while parasite reservoirs in asymptomatic carriers could hamper the progress of malaria control and eradication efforts. Therefore, active detection of infected carrier could be useful for a malaria elimination programme [10].

This study explored the prevalence of asymptomatic malaria carriers in two of the four epidemiological areas in Madagascar. Blood samples collected in 2010 from 1,244 asymptomatic pregnant women from three coastal and three highland cities were investigated for: (i) the presence of malaria parasites, (ii) the seroprevalence of anti-*P. falciparum* antibodies, (iii) the multiplicity of infection and (iv) the presence of drug resistance markers for sulphadoxine-pyrimethamine (SP) and chloroquine (CQ).

## Methods

### Study sites

**Figure 1:**
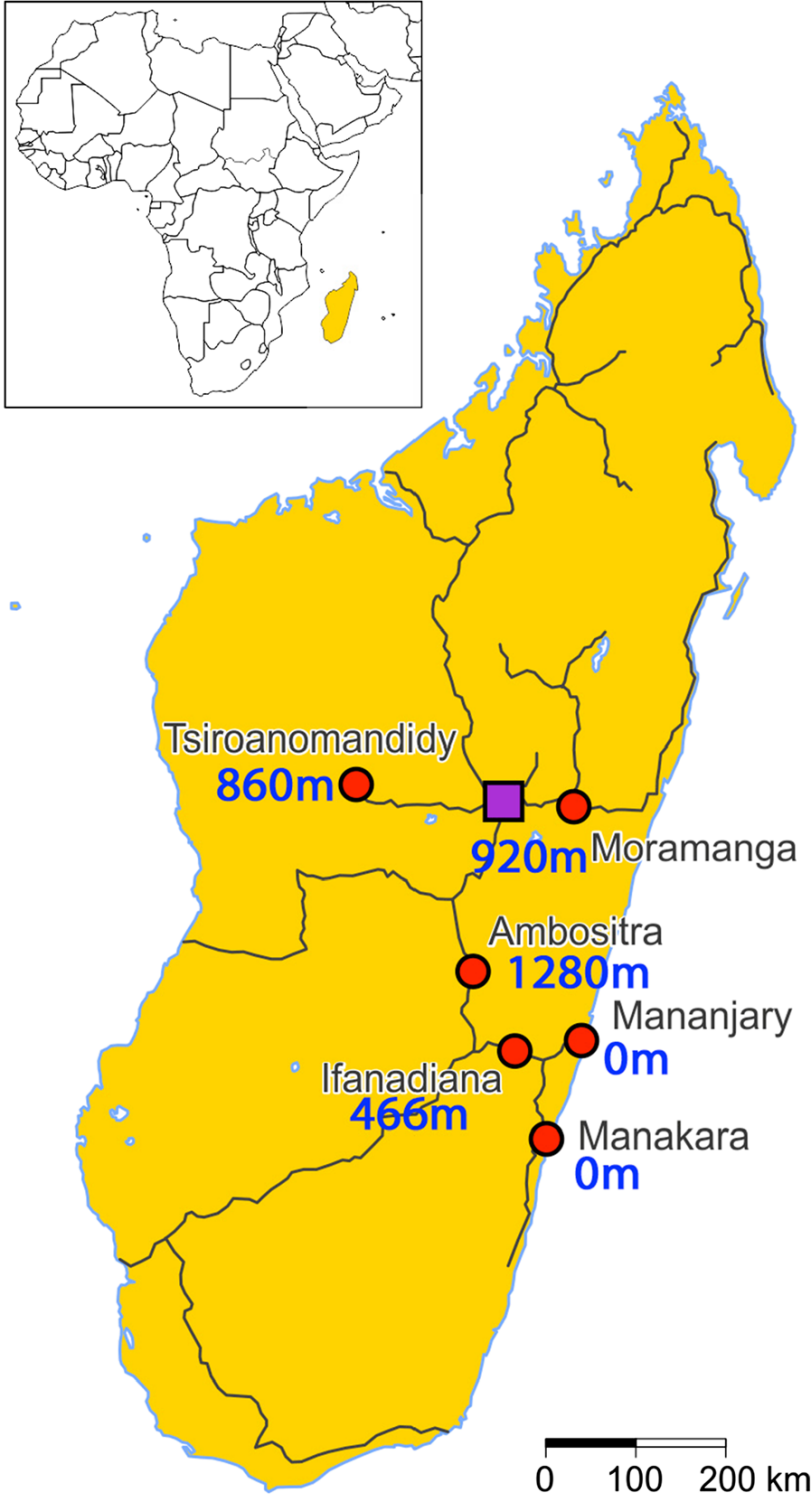
Map of Madagascar showing the six study locations with their altitude. The red circles indicate the six study locations and the purple square the capital Antananarivo. The black lines represent the main roads for long distance travel.

A cross-sectional survey was carried out between May and July 2010 in antenatal clinics in six different locations (Figure 1). The Central highlands of Madagascar are mainly on an altitude between 800 to 1,400 m above sea level. The three highland cities included in this study were Tsiroanomandidy (860 m), Moramanga (920 m), and Ambositra (1,280 m). From the central highlands the land descends over a steep fall into a ribbon of rainforest that reaches to the east coast. Mananjary and Manakara are on sea level at the east coast, and Ifanadiana is a 460 m high settlement located on the steep slope from the east coast to the highlands. The three first locations are later in this report defined as the ‘highlands’ and the three last as the ‘coast’. Mananjary and Manakara were affected by an outbreak of chikungunya 3–5 months before this study took place [11].

The study was approved by the “Comité d’éthique de la Vice Primature Chargée de la Santé Publique” from Madagascar. The study was explained to the pregnant women and their written informed consent was sought. In case of illiteracy the study was explained and approval of participation expressed through a fingerprint and signature of a witness.

### Sample collection

A venous (EDTA) blood sample was taken and the plasma was extracted by centrifugation. The supernatant was stored at −20°C for serological analysis. Five hundred microlitre of the pellet was taken and diluted with the same amount of 8 M Urea solution (v/v) that is appropriate for conserving the sample at +4°C for later DNA extraction.

### Detection of antibodies by serology

Serology for the detection of antibodies (IgG + IgA + IgM) directed against *Plasmodium falciparum* was performed by an in-house indirect immunofluorescence antibody test (IFAT). Plasma samples were diluted to 1:40 with PBS plus milk powder 3%. Twenty μL of the diluted plasma were applied to an antigen field, and were incubated for 30 min at 37°C. One positive control and one negative control were included in each series. The slides were washed three times for 5 min in PBS and dried before adding fluorescein-labelled total human anti-Ig conjugate (Sifin, Berlin, Germany) diluted at 1/200 in PBS containing Evans blue (0.1 g/L). After incubation for 30 min at 37°C in a humid chamber, the slides were again washed three times for 5 min in PBS. The slides were read under a fluorescence microscope (Zeiss) with UV-illumination at a magnification of 250. Samples fluorescing at 1/40 dilution were considered positive. An exact titer of the positive samples was not determined.

The in-house immunofluorescence test itself was manufactured using the laboratory strain 3D7 as antigen. The *Plasmodium* strain was cultured in red blood cells obtained from German Red Cross Blood Bank as described elsewhere [12]. Erythrocytes containing mature parasite forms were purified using a commercially available magnetic cell fractionation system (MACS system; Miltenyi Biotec). The purified erythrocytes were applied to glass slides with 12 fields (Thermo Scientific, Dreieich, Germany), air dried and stored at −70°C for long-term storage and subsequently at −20°C few days before usage. After thawing, the antigen slides were fixed in acetone/methanol solution (v/v) for 5 min at 4°C.

### Molecular genotyping

#### DNA extraction

DNA was extracted from 400 μL of peripheral venous blood-EDTA/8 M urea (v/v) using the QIAamp® DNA Blood mini Kit according to the manufacturer’s protocol (Qiagen).

#### Molecular detection of malarial species

Four *Plasmodium* species with human as host were assessed by real-time PCR methodology based on Fluorescence Resonance Energy Transfer technology. *P. falciparum* was detected with primers and probes targeting the *cox1* gene and *P. ovale*, *P. malariae* and *P. vivax* with primers and probes targeting the *18S rDNA* (Additional file 1).

On a LightCycler 480 (Roche), the PCR was carried out in a total volume of 20 μL containing 5 μL of extracted genomic DNA or plasmids including the gene targeted for each species as positive control. The mixture to detect *P. falciparum* contained 0.2 mM dNTPs, 3.5 mM MgCl_2_, 0.4 μM of forward primer, 0.5 μM of reverse primer, 0.15 μM each of the donor and acceptor probes, 0.25 g/l bovine serum albumin (BSA) and 0.5 U Taq polymerase (Platinium, Invitrogen). The multiplex PCR to detect *P. malariae*, *P. ovale* and *P. vivax* used three forward primers; each primer is specific of one species and one reverse primer for the three species. The mixture differed from the first one by primer concentration: 0.7 μM of forward primer for *P. malariae*, 0.5 μM of each forward primer for *P. ovale* and *P. vivax*, 0.4 μM of reverse primer.

For both PCRs, the following PCR programme was used: 2 min at 95°C; 10 touchdown repeated cycles of 5 sec at 95°C, 10 sec at 63°C to 58°C, 7 sec at 72°C; and 45 repeated cycle of 5 sec at 95°C, 10 sec at 58°C, 7 sec at 72°C. Melting analysis was performed by denaturing for 30 sec at 95°C and cooling for 2 min to 45°C followed by heating at the rate of 0.1°C/s from 45°C to 75°C.

#### Assessing multiplicity of infection of *Plasmodium falciparum*

To determine the minimal number of distinguishable clones per isolate, we genotyped 6 different neutral highly polymorphic, microsatellites *ARA2* (chr 11, Genbank accession no. X17484), *PfPK2* (chr 12, X63648), *TA60* (chr 13, AF010556), *TA81* (chr 5, AF010510), *TA87* (chr 6, AF010571), *Poly alpha* (chr 4, L18785). The multiplicity of infection (MOI) was quantified as the “highest number of fragments” for one of the microsatellites genotyped.

The six microsatellite loci were amplified by semi-nested PCR according to a previous publication [13] with few modifications. Three outer PCR duplexes were performed followed by six nested PCRs. Each outer PCR mixture (50 μL) contained 6 μL of DNA, 3 mM MgCl_2_, 200 μM dNTPs, 50 nM for each primer (4 in total for Poly-alpha and TA60, TA81 and ARA2 or PfPK2 and TA87; Additional file 1) and 1.25 U Taq polymerase (GoTaq, Promega). The sequences of all primers used are shown in Additional file 1. Cycling conditions were as follows: 2 min at 94°C; 25 repeated cycles of 30 sec at 94°C, 30 sec at 42°C, 30 sec at 40°C, 40 sec at 65°C; 2 min at 65°C. Each nested PCR mixture contained 1 μL of the outer PCR, 2.5 mM MgCl_2_, 200 μM dNTPs, 150 nM for each primer and 1 U Taq polymerase (GoTaq, Promega). Cycling conditions were as follows: 2 min at 94°C; 25 repeated cycles of 20 sec at 94°C, 20 sec at 45°C and 30 sec at 65°C; 2 min at 65°C. Amplified fragments were analysed on an ABI 3130 Genetic Analyzer and by GeneMapper® software version 4.0 (Applied Biosystems) to measure the variable length in the samples.

#### Detection of *Plasmodium falciparum* drug resistance markers by nested PCR-RFLP

For this study, the polymorphism of *pfdhfr* at codons N51I, C59R, S108N and I164L, *pfdhps* at codons A437G and K540E, *pfcrt* at codon K76T and *pfmdr1* at codon N86Y were genotyped as described previously [14]. Because we suspected low parasitaemia, the outer PCR mixtures were carried out in a total volume of 50 μL containing 6 μL of template DNA. DNA from two *P. falciparum* strains (3D7, and W2mef) was used as controls for the PCR and the digestion.

### Sample size calculations

For this study, the aim was to detect at least one pregnant woman with malaria parasites if the real prevalence was 2.5% with a 90% probability. Using binomial distribution as a proxy for a hypergeometrical distribution, the required sample size of 152 was calculated. Therefore aimed to include at least 160 women per study site. With 200 pregnant women per study site, the 90% probability to detect at least one parasitized women holds even if the real prevalence is only 2%.

### Statistical analysis

For the statistical analysis of the study univariate tests were performed. All categorical variables were analysed conducting Fisher’s exact tests. As a normal distribution of the MOI could not be assumed Mann Whitney’s U test and Kruskall Wallis test as the non-prarametric equivalent to an ANOVA were conducted for this variable. The two-sided significance level for all tests was set to 0.05 and not adjusted for multiple testing as the study was planned as a descriptive study.

## Results

Overall, 1,244 pregnant women were included into the study. In all study locations, the age distribution was right skewed with a median age of 25 years (range 12–50). Subsequently, different variables were analysed according to two age groups defined as up to 19 years and, 20 years and over. Nine out of 601 women questioned from the highlands claimed to travel within the last half of a year and only one of these travels was to a coastal location.

The proportion of primiparae was similar in the highlands and at the coast (28.4% and 30.3%, respectively). The only noteworthy mosquito protective measures that were used frequently were bed nets (70.3%). The use of bed nets was more common in the coastal cities of Mananjary (90.5%), Manakara (91.2%) and Ifanadiana (94.4%) compared to the highland cities of Tsironoamandidy (56.7%), Moramanga (67.0%) and Ambositra (21.5%). Intermittent presumptive treatment in pregnancy (IPTp) was reported by 41.3% overall. The proportion of women who had received IPTp was higher at the coast than in the highlands (p > 0.001) (Table 1).

**Table 1:** **Prevalence of primigravidae women, bed net use and uptake of IPTp at six locations in Madagascar**

	**Coast**						**Highlands**						**Total**	
	**Mananjary**		**Manakara**		**Ifanadiana**		**TDD**		**Moramanga**		**Ambositra**			
	**N**	**+ (%)**	**N**	**+ (%)**	**N**	**+ (%)**	**N**	**+ (%)**	**N**	**+ (%)**	**N**	**+ (%)**	**N**	**+ (%)**
primigravidae	188	64 (34.4)	245	58 (23.7)	178	48 (27.0)	202	63 (31.2)	196	66 (33.7)	190	49 (25.8)	1199	348 (29.0)
Bed net use	190	172 (90.5)	250	228 (91.2)	197	186 (94.4)	203	115 (56.7)	194	130 (67.0)	200	43 (21.5)	1234	874 (70.8)
IPTp/SP	190	88 (46.3)	243	96 (39.5)	196	126 (64.3)	198	95 (48.0)	191	89 (46.6)	190	5 (2.6)	1208	499 (41.3)

TDD, Tsiroanomandidy.

IPTp/SP, Intermittent Presumptive Treatment in pregnancy with sulphadoxine-pyrimethamine.

N, Number of participants who answered the questionnaire.

+, Number of primigravidae participants or participants who claimed to use bed net or IPTp/SP as prophylaxes.

### Prevalence of anti-*Plasmodium falciparum* antibodies

**Figure 2:**
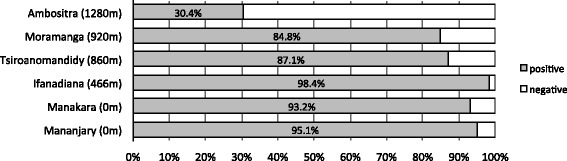
Seroprevalence of anti-*Plasmodium falciparum* antibodies in pregnant women in six locations of Madagascar. The altitude of each site is indicated in brackets. The grey bars indicate the percentage of seropositive samples and the white bar the seronegative samples.

The prevalence of antibodies directed to *P. falciparum* was determined using an IFAT for 1216 samples where serum or plasma was available. In the coastal areas, the seroprevalence was very high with 95.6% of the samples (Figure 2). The prevalence according to age categories up to 19 years and, from 20 years and over were similar with 93.7% and 95.9%, respectively. In the highlands, the seroprevalence was slightly lower with 87.1% in Tsiroanomandidy and 84.8% in Moramanga. In those two locations, the seroprevalence was also equivalent according to age groups. In Ambositra, located higher than 1,280 m above sea level, antibodies against *P. falciparum* were detected only in 30.4% of the pregnant women. The seroprevalence was lower in the pregnant group up to 19 years with 25.9% than in the group of 20 years and over with 30.4%.

### Prevalence of infection

Of all 1,242 blood samples, 157 (12.6%) were PCR-positive for *P. falciparum*, nine for *P. malariae*, one for *P. ovale* and one for *P. vivax*; six mixed infections were found (Table 2). With 14.8% the mean of infected pregnant women was higher at the coast than at highland locations (9.2%, p < 0.001). In Ifanadiana, which is situated 466 m above sea level on the ascend from the coastal study sites to the highlands, prevalence of 9.6% was found, slightly lower than two of the three sites in the highlands: Tsironomandidy (10.3%) and Moramanga (13.6%).

**Table 2:** **Prevalence of malaria parasites detected by realtime-PCR in pregnant women at six locations of Madagascar**

	**Coast**						**Highlands**						**Total (N = 1242)**	
	**Mananjary (N = 195)**		**Manakara (N = 249)**		**Ifanadiana (N = 197)**		**TDD (N = 203)**		**Moramanga (N = 198)**		**Ambositra (N = 200)**			
**Number of samples**	**n**	**%**	**n**	**%**	**n**	**%**	**n**	**%**	**n**	**%**	**n**	**%**	**n**	**%**
*P. falciparum*	25	12.8	51	20.5	19	9.6	21	10.3	27	13.6	14	7	157	12.6
*P. malariae*	0	-	5	2	3	1.5	1	0.5	0	-	0	-	9	0.7
*P. ovale*	0	-	0	-	1	0.5	0	-	0	-	0	-	1	0.1
*P. vivax*	0	-	0	-	0	-	1	0.5	0	-	0	-	1	0.1
*Pf* + *Pm*	0	-	3	1.2	1	0.5	1	0.5	0	-	0	-	5	0.4
*Pm* + *Po*	0	-	0	-	1	0.5	0	-	0	-	0	-	1	0.1

TDD, Tsiroanomandidy.

N, Number of EDTA-blood samples collected.

n, number of positive samples for *Plasmodium* species detection by realtime-PCR.

At the coast, the prevalence of infection was lower in pregnant women using prophylaxis than in women without prophylaxis. With IPTp, the prevalence was 14.0% vs. 18.9% and with bed net use; it was 14.2% vs. 21.6%, respectively (Table 3). For the analysis of the MOI and for the detection of drug resistant markers, only the 152 samples that were mono-infected by *P. falciparum* were included.

**Table 3:** **Effect of prevention used against malaria among pregnant women in Malagasy coast and highlands**

		**Coast**				**Highlands**			
		**N**	***Pf +*** **(%)**	**OR [CI 95%]**	p*	**N**	***Pf +*** **(%)**	**OR [CI 95%]**	p*
Gravidea	1	437	62 (14.2)	0.95 [0.57-1.58]	0.474	410	38 (9.3)	1.45 [0.84-2.52]	0.119
	≥2	170	23 (13.5)			178	23 (12.9)		
Bed net use	no	51	11 (21.6)	0.57 [0.28-1.16]	0.09	309	32 (10.4)	0.93 [0.55-1.59]	0.452
	yes	584	79 (13.5)			288	28 (9.7)		
IPTp/SP	no	317	60 (18.9)	0.43 [0.26-0.69]	>0.001	390	37 (9.5)	1.26 [0.72-2.20]	0.253
	yes	310	28 (9.0)			189	22 (11.6)		

*Pf+*, Number of positive samples with *Plasmodium falciparum* by realtime-PCR.

OR, odd ratio.

IPTp/SP, Intermittent Presumptive Treatment in pregnancy with sulphadoxine-pyrimethamine.

*Fisher’s exact test.

### Multiplicity of infection

**Figure 3:**
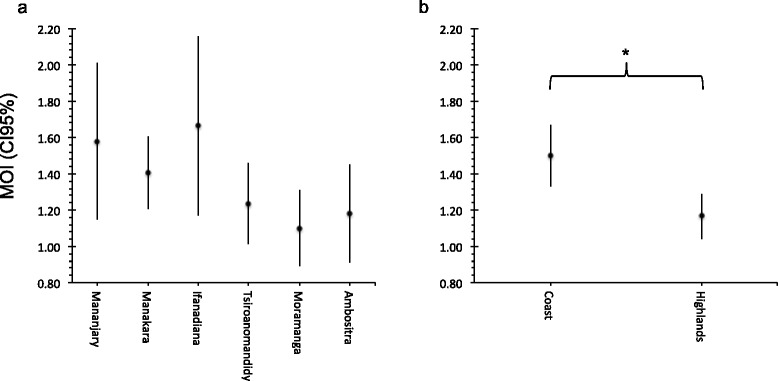
Multiplicity of infection in different locations in Madagascar. The mean of the multiplicity of infection (MOI) and its 95% confidence interval (CI 95%) at each study sites **(a)** and between costal and highland study sites in Madagascar **(b)**. For each samples, the MOI was determined by the highest number of clones found by one of the six microsatellites genotyped. The MOI was significantly higher in the coast than in the highlands (*Mann Whitney test, *p* = 0.005) but no significant difference was found between each site (Kruskal Wallis test, *p* = 0.065).

The six neutral microsatellites genotyped in this study were highly polymorphic in the six locations with a number of alleles from nine for ARA2 and TA60 to 14 for PfPK2. The MOI could be determined for 116/152 samples by at least one microsatellite. The highest maximal number of different parasite clones detected in one pregnant woman was four in a costal location (Mananjary) with PfPk2. The average number of different clones found in infected pregnant woman was 1.4 and it was higher at the coast level (1.5, 95% CI: 1.3-1.7) than in the highlands (1.2, 95% CI: 1.0-1.3; p = 0.005) (Figure 3). The prevalence of *P. falciparum* monoclonal infection was 61.8% at the coast against 85.4% in the highlands (p = 0.005) (Table 4). According to the group of age, the MOI was higher in the group of pregnant women up to 19 years with 1.55 than in the one from 20 and over with 1.3 (Table 5). The MOI was slightly higher in the group of pregnant women who didn’t use IPTp than in the one who used the chemoprophylaxis in the highlands (1.21 vs. 1.06) and at the coast (1.52 vs. 1.44) (Table 5). Furthermore, the use of bed net was significantly associated with the reduction of MOI (2 vs. 1.43, p = 0.028) in the coast level (Table 5).

**Table 4:** **Polyclonality of malaria infection in six locations in Madagascar**

	**Coast**						**Highlands**					
**Number of clones**	**Mananjary (N = 19)**		**Manakara (N = 37)**		**Ifanadiana (N = 12)**		**TDD (N = 17)**		**Moramanga (N = 20)**		**Ambositra (N = 11)**	
	**n**	**%**	**n**	**%**	**n**	**%**	**n**	**%**	**n**	**%**	**n**	**%**
1	12	63.2	24	64.9	6	50	13	76.5	19	95	9	81.8
2	4	21.1	11	29.7	4	33.3	4	23.5	0	-	2	18.2
3	2	10.5	2	5.4	2	16.7	0	-	1	5	0	-
4	1	5.3	0	-	0	-	0	-	0	-	0	-
MOI [CI 95%]	1.6 [1.1-2.0]		1.4 [1.2-1.6]		1.7 [1.2-2.2]		1.2 [1.0-1.5]		1.1 [0.9-1.3]		1.2 [0.9 - 1.5]	

TDD, Tsiroanomandidy.

MOI, multiplicity of infection.

N, Number of *Plasmodium falicparum* samples able to be genotyped for microsatellite loci.

**Table 5:** **Effect of prevention used against malaria among pregnant women on the MOI in Malagasy coast and highlands**

		**Coast**			**Highlands**		
		**N**	**MOI**	p*	**N**	**MOI**	p*
Age	<20	20	1.7	0.142	11	1.27	0.418
	≥20	48	1.42		34	1.15	
Bed net use	no	9	2	0.028	28	1.14	0.605
	yes	58	1.43		19	1.21	
IPTp/SP	no	48	1.52	0.71	29	1.21	0.229
	yes	18	1.44		18	1.01	

MOI, multiplicity of infection.

IPTp/SP, Intermittent Presumptive Treatment in pregnancy with sulphadoxine-pyrimethamine.

*Mann Whitney test.

### Prevalence of drug resistance markers

**Figure 4:**
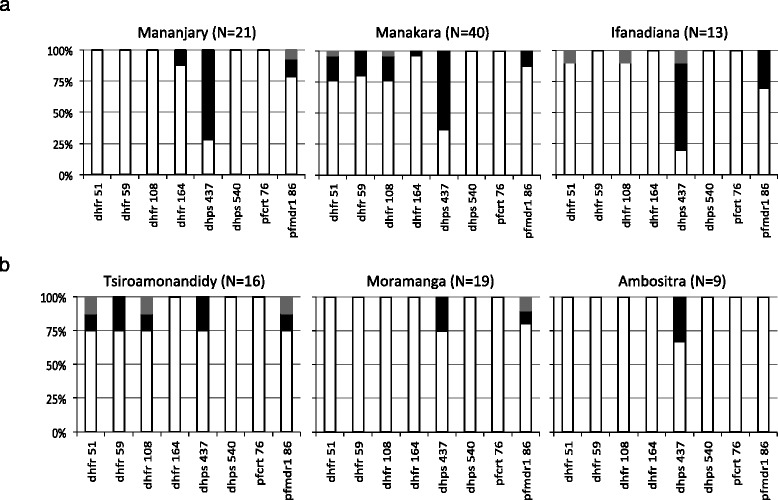
Distribution of *Plasmodium falciparum* drug resistance alleles in the coast **(a)** and in highlands **(b)** of Madagascar. *pfcrt* and *pfmdr1* mutations are associated with chloroquine resistance, *dhfr* and *dhps* mutaions with sulphadoxine-pyrimethamine resistance. The white bars indicate the proportion of samples with wild-type allele, the black bars the proportion samples with mutant allele and the grey bars the proportion samples with both alleles. In brackets, N represents the number of *Plasmodium falciparum* samples able to be genotyped by PCR-RFLP for each study sites.

Overall 152 isolates were screened for eight drug resistance markers against SP (*dhfr* N51I, C59R, S108N, I164L and *dhps* A437G, K540E) and CQ (*pfcrt* K76T and *pfmdr1* N86Y); 118 (77.6%) samples were successful genotyped for at least one of the eight markers. Figure 4 gives an overview of the proportional distributions of wild type, mutant and mixed type in the different study locations. The allele *dhfr* triple mutant 51I, 59R, 108N were polymorphic only in Manakara and Tsiroanomandidy with a prevalence of 20% (6/30) and 25% (2/8), respectively. The allele *dhfr* 164L was genotyped in three samples from the coast. The allelic frequency of *dhps* 437G was variable among the different locations (p = 0.02). With 71.6%, the prevalence of the mutant was higher at the coast compared to the highlands with 27.3% (p < 0.001). Overall positive samples for *dhfr* and *dhps* genotyping (N = 93), 6 (6.5%) samples were carrying the quadrupule mutant *dhfr* 51I, 59R, 108N + *dhps* 437G. Very few samples carried mutations on *pfmdr1* genes and the wild type allele of *pfcrt* K76 and *dhps* K540 were fixed in all location.

## Discussion

This study on healthy pregnant women demonstrates that *P. falciparum* parasites can be found in resident populations of Madagascan lowlands and highlands. As classical microscopy has sensitivity limitations when parasite levels are low [15], the epidemiological analysis was based on PCR.

### Prevalence of infection and immunity

Even in the highest study location, 1,200 m above sea level, 7% of healthy participants were *P. falciparum* positive. The permanent presence of *P. falciparum* in highland populations with very low levels of parasitaemia may maintain some level of partial immunity in the population indicated by the high seroprevalence in the area around 900 m above sea level (with 87.1% in Tsiroanomandidy and 84.8% in Moramanga). However, the weak clinical immunity together with a relevant parasite reservoir could initiate malaria epidemics like in Ambositra (1200 m above sea level) where the seroprevalence in pregnant women were around 30% and seems to be contracted in older category of age. Indeed, the malaria epidemics in the highlands between 1986 and 1988 were devastating with around 70,000 to 100,000 deaths every year [2]. Therefore, a significant frequency of submicroscopic malaria parasite carriage has to be considered for the planning of control measures [16]. Indeed, efficient transmission to mosquitoes has been demonstrated also at submicroscopic gametocyte densities [1,17].

### Control of malaria in the highlands

In this study, the use of bed nets or chemoprophylaxis was associated with a reduction of infections in participants from the coast. Currently, malaria control in the Malagasy highlands is mainly based on indoor insecticide spraying [7]. It cannot be excluded that parasites detected in highland locations were acquired at the coast and then imported as it occurs in the capital Antananarivo [18]. Nevertheless, the data indicate a certain level of transmission in the central highlands because only one of 601 women from the highlands travelled to a coastal location within the last half of year. Transmission in the highlands is supported by entomological surveys that have shown the occurrence of malaria vectors, such as *Anopheles gambiae*, at altitudes above 1,200 m [19].

### Mutations providing resistance against chloroquine and sulphadoxine-pyrimethamine

An unusual haplotype carrying the single nucleotide polymorphism (SNP) I164L on the *dhfr* gene involved in pyrimethamine resistance was previously found only in Madagascar [20]. This SNP was detected in three samples from pregnant woman out of 70 genotyped but never in association with the triple mutation *dhfr* 51I, 59R, 108N that is associated with a high resistance of *P. falciparum* against antifolate drugs [21]. On the other hand, four samples carrying the quadruple mutant *dhps* 437G + *dhfr* 51I, 59R, 108N in Manakara (11.8%) and two samples in Tsiroanomandidy (15.4%) were found. Accumulation of mutations in these two genes is associated with *in vivo* SP resistance [22]. Similar findings were previously reported from samples collected in Madagascar from 2006 to 2008 [20]. Even if these findings are not alarming, a constant monitoring of SP resistance is advisable because SP is the drug of choice for malaria prevention in pregnancy.

Prevalence of the principal resistance marker for CQ *pfcrt* 76T was low in Madagascar compared to continental Africa [23]. However, *in vivo* resistance to CQ in Madagascar that is not associated with the mutation *pfcrt* 76T but with the mutation *pfmdr1* 86Y was found and perhaps yet unknown mechanism were involved [24,25]. In our study we found that the prevalence of *pfmdr1* 86Y was low compared to previous data [20,23-25] maybe because of the change of the first line policy from CQ to artemisinin combination therapy (ACT). Nevertheless, a recent publication [14] has shown that the wild-type *pfmdr1* N86 SNP is associated with recrudescence of malaria infection after artemether-lumefantrine treatment in Benin. Studies on emerging ACT resistance can provide more information for the guideline of malaria treatment in Madagascar.

### Multiplicity of infection

Lower numbers of different parasite strains were found in infected women from the highlands compared to the coast. This was expected, as multiplicity is a function of transmission likelihood. The average of two to four *P. falciparum* strains in the two coastal areas are in accordance with what was described in areas of moderate to high endemicity [26]. Low parasite diversity combined with low endemicity are factors associated with emergence of malaria drug resistance like in South America or South-East Asia [27]. The findings presented here are based on a relative homogeneous subgroup of the population at risk. Similar studies including children and also other adults are needed to get more information on asymptomatic malaria parasite carriers at different altitudes.

### Effect of preventive measures on parasite carriage

In this study, the use of chemoprophylaxis (IPTp/SP) and vector control (bed net) was assessed among pregnant women from costal and highland locations of Madagascar. The use of those preventive measures against malaria was associated with the reduction of around 50% *falciparum* parasite carriage and also with the reduction of the number of clones of parasites in one infection, especially in the coastal area where the population are targeted by the mass distribution of long-lasting insecticidal nets and IPTp.

The findings of this study are in accordance with the conclusion of the nationwide evaluation of malaria infection and coverage of malaria control interventions in Madagascar, which took place in 2012–2013 [28]. Malaria control interventions should consider the whole population from the endemic area like the coast but also from the highlands.

## Conclusion

This study demonstrated that asymptomatic malaria parasite carriers occur in Madagascar up to altitudes of 1,200 m with seroprevalences under 50%. The constellation of an existing reservoir and low immunity in the population is prone for malaria epidemics. The prevalence of drug resistance markers is still not alarming compared to some areas in the African mainland but monitoring might be valuable in Madagascar where malarial parasites seem to have different drug resistance mechanism than in other areas. As the Malagasy National Malaria Control Programme is moving toward the elimination of malaria in the highlands, monitoring of the existence of parasites in asymptomatic individuals becomes more important. More sensitive technology like PCR based diagnostic for the detection of submicroscopic parasitemia provides useful information for malaria control.

## Additional file

Additional file 1:
**Primers and probes used to determine** ***Plasmodium species*** **by realtime-PCR and to genotype six neutral** ***P. falciparum*** **microsatellites.**
